# Novel Biocompatible Zr-Based Alloy with Low Young’s Modulus and Magnetic Susceptibility for Biomedical Implants

**DOI:** 10.3390/ma13225130

**Published:** 2020-11-13

**Authors:** Renhao Xue, Dong Wang, Dawei Yang, Ligang Zhang, Xiaoning Xu, Libin Liu, Di Wu

**Affiliations:** 1 School of Material Science and Engineering, Central South University, Changsha 410083, China; xuerenhao@csu.edu.cn (R.X.); 183112099@csu.edu.cn (D.W.); ligangzhang@csu.edu.cn (L.Z.); xxning@csu.edu.cn (X.X.); 2 State Key Laboratory of Powder Metallurgy, Central South University, Changsha 410083, China; 3 Centre for Medical Genetics and School of Life Science, Central South University, Changsha 410008, China; yangdawei@sklmg.edu.cn; 4 School of Metallurgy and Environment, Central South University, Changsha 410083, China

**Keywords:** Zr-Nb-Ti alloys, Young’s modulus, magnetic susceptibility, corrosion behavior, cytocompatibility, blood compatibility

## Abstract

The microstructure, mechanical properties, magnetic susceptibility, electrochemical corrosion performance, in vitro cell compatibility and blood consistency of Zr-16Nb-xTi (x = 0, 4, 8, 12 and 16 wt.%) materials were investigated as potential materials for biomedical implants. X-ray diffraction (XRD) and Transmission electron microscopy (TEM) analyses revealed the secondary phase martensite α’ formed during the quenching process. The phase composition contained metastable β and martensite α’, resulting from Ti addition. These phase constitutions were the main causes of a low Young’s modulus and magnetic susceptibility. The in vitro cytocompatibility analysis illustrated that the MG63 cells maintained high activity (from 91% to 97%) after culturing in Zr-16Nb-xTi extraction media for 12 days due to the high internal biocompatibility of Zr, Nb and Ti elements, as well as the optimal corrosion resistance of Zr-16Nb-xTi. On the basis of Inductively coupled plasma optical emission spectrometry (ICP-OES) ion release studies, the concentration of Zr, Nb and Ti was noted to reach the equipment detective limit of 0.001 mg/L, which was much lower than pure Ti. With respect to the corrosion behavior in Hank’s solution, Zr-16Nb-16Ti displayed superior properties, possessing the lowest corrosion current density and widest passivation region, attributed to the addition of Ti. The blood compatibility test illustrated that the Zr-16Nb-xTi materials were nonhemolytic, and the platelets maintained a spherical shape, with no aggregation or activation on Zr-16Nb-xTi. Overall, Ti addition has obvious effects on the developed Zr-16Nb-xTi alloys, and Zr-16Nb-4Ti exhibited low magnetic susceptibility, low modulus, good biocompatibility and proper corrosion properties, demonstrating the potential of use as implant biomaterials.

## 1. Introduction

Ascribed to the growing older population, elderly people are at a high risk of hard tissue failure. Kurtz et al. [1] mentioned that in 2020, the number of total hip replacements would reach 384,000 and increase to 572,000 by 2030. These undoubtedly put high demands on the quantity and quality of implant materials [2].

Metals and their alloys are vital for use in biomedical materials because of their high strength and processability. Alloys such as Ti-based materials, 316 L stainless steel and Co-Cr-Mo alloy are widely used as biomedical materials [3]. Most of these alloys, such as titanium alloys (55–115 GPa) [4], 316L SS (200–210 GPa) [4,5] and Co-Cr alloys (210–253 GPa) [4,5], present a larger Young’s modulus than human bone (10–30 GPa) [6], which results in a “stress shielding” effect and further implant failure [7,8]. Magnetic resonance imaging (MRI) generally serves as a significant diagnostic instrument for orthopedics [9]. To obtain high-quality images, MRI facilities need to enhance magnetic field intensity, which may lead to artifacts if there are metallic implants in the human body, resulting in an incorrect diagnosis. Though Ti-based alloys possess lower magnetic susceptibility than 316L SS and Co-Cr-Mo alloys, it has been reported that Ti-based alloys would cause image distortion during MRI diagnosis [10,11]. More importantly, metallic implant materials must be biocompatible, corrosion-resistant and tissue-compatible [12,13]. For the past few years, Ti and Ti-based alloys have been confirmed to largely meet these requirements [14]. Ti-6Al-4V has been the most widely used material for this purpose. Owing to the development of in vitro and in vivo techniques, a growing number of studies have identified the harmful effects of vanadium [15] in elemental and oxide states. For instance, an extremely high release of proinflammatory and osteolytic mediators occurred when bone marrow cells were cultured in Ti-6Al-4V, resulting in the aseptic loosening of prostheses [16].

Three essential requirements need to be fulfilled in implant biomaterials: an appropriate Young’s modulus, magnetic susceptibility and excellent biocompatibility [17,18,19]. Compared with pure Ti, Zr exhibits a lower modulus of 95–100 GPa, exhibiting the possibility to develop Zr alloys with a lower modulus to alleviate the “stress shielding” effect. Zr possesses a considerably lower magnetic susceptibility ($\chi_{Zr}$ = 1.32 × 10^−6^ cm^3^/g) than Ti ($\chi_{Ti}$ = 3.15 × 10^−6^ cm^3^/g) [20]. Zhou et al [21]. reported that Zr-based alloys have superior biocompatibility compared with traditional biomedical alloys.

The above information infers that among all potential alloys, Zr-based alloys exhibit the proper capacity to deal with the existing problem in biomedical alloys. Previous studies have indicated that Zr obstructs martensite formation in Ti-Nb alloys by reducing the Ms temperature [22,23], and Zr can be visualized as a β stabilizing element in Ti-Nb systems. However, investigations on Zr-based alloys in the field of biomaterials are lacking, especially considering the effect of Ti addition in Zr-based alloys. 

The most commonly used β stabilizing elements are V, Fe, Mo, Ta, Cr, Nb, Ni, Cu. Fe, Ta, Nb and Mo, which have been observed to be biocompatible. Eisenbarth et al. [12]. observed that Mo has a negative effect on cell proliferation, mitochondrial activity and cell volume after contact for seven days. On the contrary, the activity and volume of the cells enhanced in contact with Ti, Nb and Ta. Kondo et al. illustrated that Nb content in the Zr-Nb binary system has an obvious effect on the phase constitution of Zr alloys. For Nb (wt.%) < 6%, α’ was observed as the main phase in the Zr-Nb alloys. For Nb (wt.%) > 6%, a ω phase was subsequently observed. A distinct ω phase was observed for Zr-9Nb, whereas a β + ω phase existed in the Nb range of 12–20 (wt.%) [24]. According to Nomura’s study [9,25,26], the phase constitution has a significant effect on magnetic susceptibility in Zr alloys and decreases in the following sequence: $\chi_{\beta }>\chi_{\alpha^{\prime }}>\chi_{\omega }$. Thus, Zr alloys containing an α’ martensite phase have been proposed as optimal candidates for implant biomaterials and use in MRI.

Based on the literature, to meet the aforementioned essential requirements, the ideal phase constitution of Zr alloys should be α’ + β to hinder the formation of a brittle and high-modulus ω phase. Zr-Nb systems alloyed by Ti were chosen to enhance the strength and control phase transition (Ti can be used as an α stabilizing element to expand the α’ + β phase range). In our experiment, when the Zr-Nb system had 16 wt.% Nb, the main phase was β and there were considerably few α’ martensite, which led to a low Young’s modulus. On adding Ti, α’ martensite could be obviously observed. Ti addition affected α’ phase amounts, and we acquired an appropriate α’ phase constitution under a proper Ti content, leading to low magnetic susceptibility, and no ω phase was detected to obtain optimal combination properties.

Consequently, we chose the as-cast Zr-16Nb-xTi (x = 0, 4, 8, 12 and 16, wt.%) to explore the Ti addition effects on the Young’s modulus, magnetic susceptibility and biocompatibility. We found the optimal combination of mechanical properties, magnetic susceptibility and biocompatibility of Zr-16Nb-xTi for potential implant alloys.

## 2. Materials and Methods

### 2.1. Materials Preparation

Zr-16Nb-xTi (x = 0, 4, 8, 12, 16, wt.%) alloys were prepared from high-purity Zr (≥99.99 wt.%), Nb (≥99.99 wt.%) and Ti (≥99.99 wt.%) using a nonconsumable arc-melting furnace under an Ar atmosphere. For an adequate mixing and reaction of the alloys in a molten state, the melting temperature was controlled at 3000 °C. Each alloy ingot was kept for 80 s. The molten ingots were then directly cooled in water-cooled copper melting pots, which maintained room temperature by running water. The cooling time was maintained at approximately 500 s. Each ingot was remelted six times by inversion to improve its chemical homogeneity.

Table 1 illustrates the chemical compositions of prepared Zr-16Nb-xTi alloys, which were determined by energy dispersive spectrometry (EDS).

### 2.2. Microstructure Characterization

X-ray diffraction (XRD), Rigaku D/Max 2500 diffractometer with Cu-Kα radiation (Rigaku, Tokyo, Japan) was used to identify the phase constitution of the Zr-16Nb-xTi alloys. The accelerating voltage and current used were 40 kV and 250 mA, respectively. Transmission electron microscopy (TEM) was employed to examine the microstructure of the Zr-16Nb-xTi alloys. The Tecnai G2 F20 transmission electron microscope (FEI Company, Hillsboro, Oregon, USA) was used to observe the microstructure of the Zr-16Nb-xTi alloys at 200 kV.

### 2.3. Mechanical Properties

Tensile tests were performed with an initial strain rate of 3.33 × 10^−2^ mm/s using the Instron 3369 mechanical testing system (Instron®, Norwood, MA, USA) at room temperature to determine the mechanical properties. An extensometer was used for accurate strain measurement. The dimensions of the tensile samples were M8 × Φ4 (GB4338-1995). Three duplicate specimens were tested from each alloy. The 0.2% offset yield strength (YS) and ultimate tensile strength (UTS) were obtained from the stress–strain curves. The Young’s modulus was calculated as the straight-line slope from the stress–strain curves, and an average of the three values was reported.

### 2.4. Electrochemical Corrosion Measurements

The electrochemical corrosion test was carried out using a three-electrode system at 37.0 ± 0.5 °C, controlled using a water bath. Saturated calomel electrodes (SCEs) acted as reference electrodes, and palladium foil was used as counter electrodes. Hanks’ balanced salt solution (HBSS) was used as an electrolyte with pH 7.4. The electrochemical properties of the Co-Cr-Mo alloy, 316L stainless steel (316L SS) and Ti-6Al-4V alloy were characterized under the same conditions for reference. Each specimen open-circuit potential (OCP) was continuously monitored for 4000–6000 s in Hanks’ balanced salt solution until it became stable, followed by potentiodynamic polarization measurements. The polarization curve was measured at potentials ranging from −1.0 to 2.0 V (vs. OCP), and the scan rate was 2 mV·s^−1^.

The corrosion current density (*I*_corr_) and corrosion potential (*E*_corr_) were obtained by employing the Tafel extrapolation method.

### 2.5. Static Immersion Test

Conforming to the ASTM-G31-72 [27] specification, the static immersion test was performed in Hank’s simulated body fluid (HSBF). The samples were cast with dimensions 10 × 10 × 1 mm^3^, followed by immersion in 48 mL HSBF solution for 30 days at an unchanging temperature of 37.0 ± 0.5 °C. Inductively coupled plasma optical emission spectrometry (ICP-OES, SPECTROBLUE SOP, SPECTRO Analytical Instruments GmbH, Kleve, Germany) was used to detect the concentration of the Zr, Ti and Nb ions in the test solutions.

### 2.6. Cell Experiments

The cytocompatibility of the Zr-16Nb-xTi alloys was assessed using human osteoblast-like cells (MG 63). The MG 63 cell line was obtained from the Center for Medical Genetics and School of Life Science, Central South University. All cells were executed according to the procedure approved by the Institutional Cell Care and Use Committee at Central South University.

MG63 cells were cultivated in the minimum essential medium (MEM), including 10% fetal bovine serum (FBS), 100 μg mL^−1^ streptomycin and 100 U mL^−1^ penicillin. These were subsequently cultivated in a cell incubator with a humidified atmosphere of 5% CO_2_ at 37 °C. Every three days throughout the duration of the cell experiments, the cultivated medium was changed. The cell viability of the Zr-16Nb-xTi alloys was evaluated using an indirect method based on ISO 10993-12:2007 [28].

Extracts of the Zr-16Nb-xTi alloys were acquired by employing the serum-free MEM for the extraction medium using 1 mL of the medium and conducting the extraction for 72 h at 37 °C with 3 cm^2^ of each sample. The cell culture medium was employed as the negative control, with the common bioalloys Ti-6Al-4V, Co-Cr-Mo alloy and 316L SS used as the control material. The MEM, including 10% dimethylsulfoxide (DMSO), was used as a positive control. The cells were initially seeded in 96-well plates at a density of 4 × 10^3^ cells per 100 μL of the medium. Later, the cells were cultured for 24 h to obtain the cell attachment, followed by the substitution of the culture media by the extracts acquired from the selected samples, incubated for 4, 8 and 12 days.

After the cultivation period, each well was placed with 10 μL of 3-(4,5-dimethylthiazol-2-yl)-2,5-diphenyltetrazolium bromide (MTT, 5 mg mL^−1^). The well plates were cultured with MTT for 4 h in a dark environment. Later, in the incubator, each well was added with 100 μL of formazan solubilization solution (10% sodium dodecyl sulfate (SDS) in 0.01 M HCl). The spectrophotometrical absorbance of the product in each well was measured via a microplate reader (Bio-Tek, Thermo Fisher Scientific, Waltham, Massachusetts, USA) at 570 nm with a reference wavelength of 630 nm.

The MG 63 cells were cultivated in the extraction media of the specimens for 7 days with a density of 4 × 10^3^ cells per 100 μL of the medium in 96-well plates to assess the alkaline phosphatase (ALP) activity. The culture medium was removed from each well and 100 μL of 1% Triton X-100 was added to acquire the cell lysates. As ALP hydrolyzes phenylphosphate to phenol and phosphate at pH 10, the corresponding ALP activity was measured. The phenol reacted with 4-aminoantipyrine in the existence of potassium ferricyanide, forming a red-colored quinone compound. The activity of ALP was proportional to the absorbance. The reaction was conducted for 15 min at a temperature of 37 °C. The reaction product’s absorbance was measured at 545 nm via a microplate reader (Bio-Tek).

The ALP activity of the selected samples was displayed as the percentage of the negative control.

### 2.7. Hemolysis Ratio Test

After mechanical polishing and ultrasonic cleaning, the test specimens were placed in centrifugal tubes, followed by the addition of 10 mL of normal saline (NS) in the tubes for the negative control group. Ten milliliters of deionized water was added to the positive control group. The venous blood was collected from the healthy adults and anticoagulated with 3.2% sodium citrate. The anticoagulated blood was mixed with normal saline at a 4:5 ratio (diluted blood), and 0.2 mL of diluted blood was added to each tube. The tubes were shaken gently and evenly to ensure sufficient contact between the material and blood, followed by placement in a 37 °C environment for 60 min. The tube liquid was centrifuged at 3000 r/min for 5 min, and the absorbance (OD) value of the supernatant was detected at 545 nm using a UW spectrophotometer (Thermo Fisher Scientific, Waltham, MA, USA).

### 2.8. Platelet Adhesion Test

Platelet-rich plasma was acquired from anticoagulated whole blood centrifuged at a speed of 1000 r/min for 10 min. The test samples were immersed in the plasma at a temperature of 37 ℃ for 1 h, followed by rinsing with normal saline for 2 min. Afterwards, 2.5% glutaraldehyde solution was used for immobilization at room temperature for 60 min. The samples were subsequently dehydrated by gradually adding alcohol at 10 min intervals. The sample surface was sprayed with gold after drying, and scanning electron microscopy analysis (ZEISS EVO MA10, 20KV, Zeiss, Oberkochen, Germany) was performed to observe the platelet adhesion on the surface.

### 2.9. Magnetic Susceptibility (χ) Measurement

The magnetic properties of the Zr-16Nb-xTi alloys were measured by employing a SQUID-VSM device (superconducting quantum interference device-vibrating sample magnetometer, Quantum Design, San Diego, CA, USA) at room temperature. The specimen magnetization (M) and applied magnetic field (H) were recorded. By taking the magnetization (M) as the *y*-axis and magnetic field (H) as the *x*-axis, the slope of the obtained magnetization curve was denoted as the magnetic susceptibility (χ). χ=M/H.

An applied magnetic field (H) was set from −30,000 Oe to +30,000 Oe, which is consistent with the magnetic field used for clinical MRI detection (3T).

### 2.10. Statistical Analysis

Statistical analysis was conducted by SPSS 18.0 software (18.0 version, IBM, Chicago, IL, USA), one-way ANOVA (18.0 version, IBM, Chicago, IL, USA), followed by a post hoc Tukey’s test for analyzing the difference among the groups. The statistical significance was set at *p* < 0.05.

## 3. Results

### 3.1. Initial Microstructure

As the Zr-16Nb-xTi alloys have a similar secondary phase (with the only exception that Zr-16Nb has less secondary phase compared to the other samples), dark-field TEM images and their corresponding selected area electron diffraction (SAED) profile were chosen to analyze the precipitated phase.

**Figure 1:**
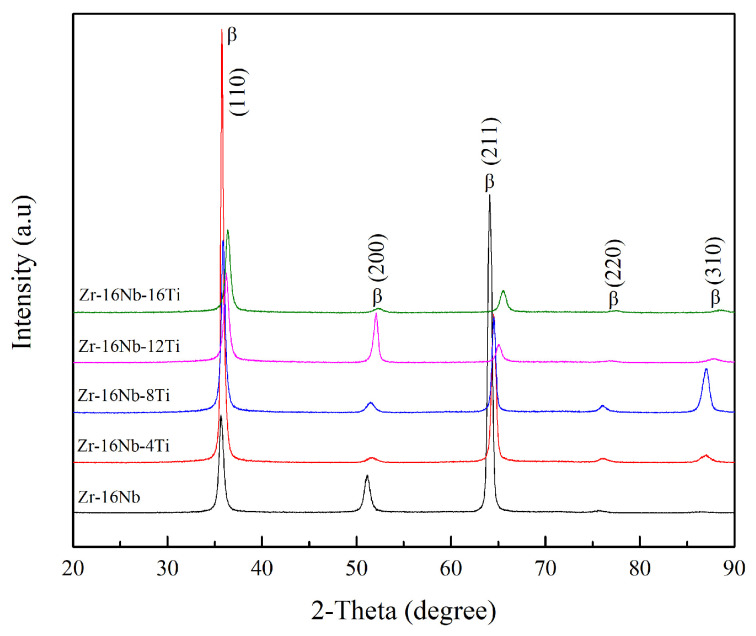
XRD patterns of the Zr-16Nb-xTi alloys.

**Figure 2:**
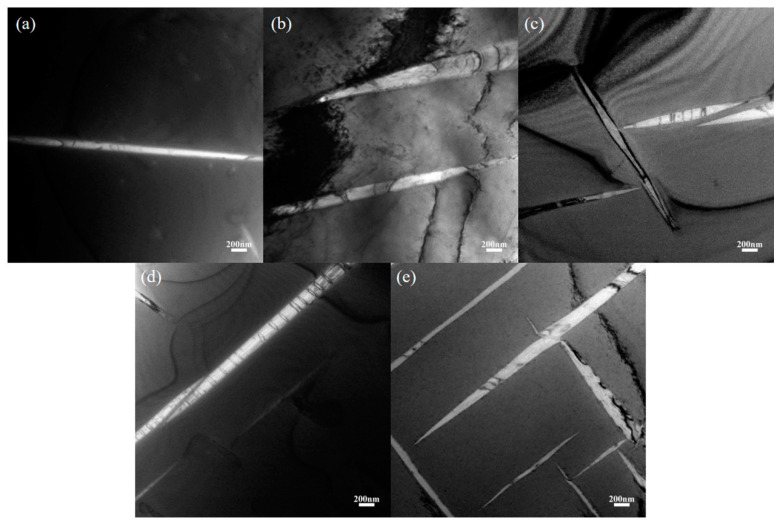
Dark-field TEM images of the martensite phase morphology of the Zr-16Nb-xTi alloys based on different areas: (**a**) Zr-16Nb; (**b**) Zr-16Nb-4Ti; (**c**) Zr-16Nb-8Ti; (**d**) Zr-16Nb-12Ti; (**e**) Zr-16Nb-16Ti.

**Figure 3:**
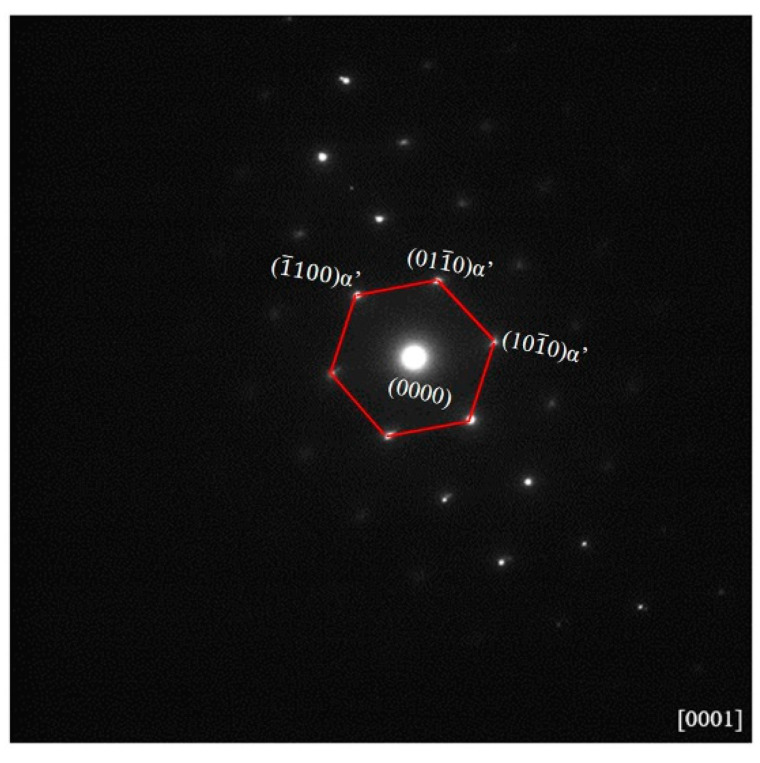
α’ martensite phase selected area electron diffraction (SAED) patterns of the Zr-16Nb-xTi alloys.

**Figure 4:**
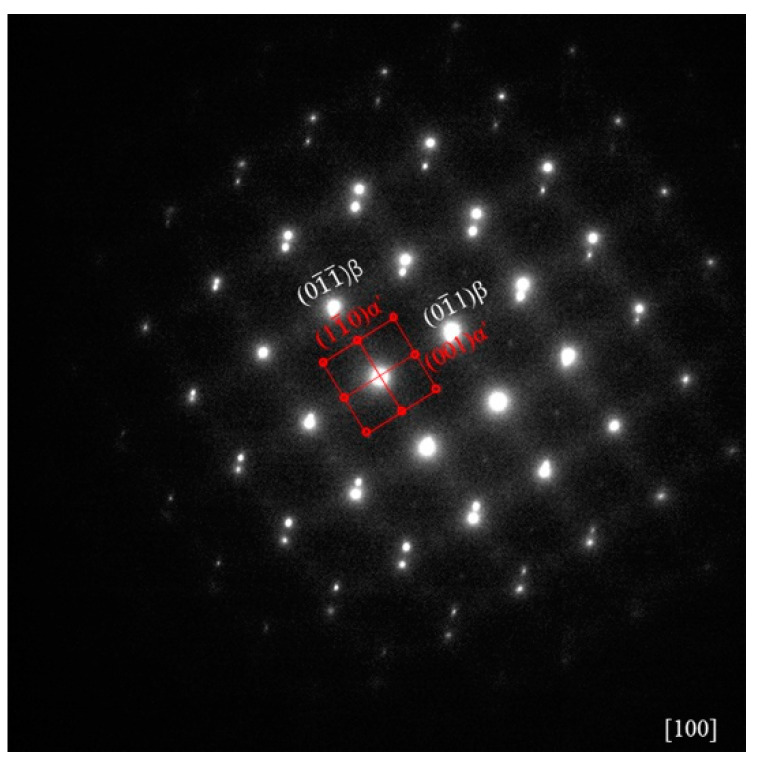
β and α’ martensite phase selected area electron diffraction (SAED) patterns of the Zr-16Nb-xTi alloys.

Figure 1 illustrates the as-cast Zr-16Nb-xTi alloy XRD patterns. Only the β phase is identified. The dark-field TEM images demonstrate an acicular phase in these alloys. Figure 2 displays phase morphology of the Zr-16Nb-xTi alloys. The SAED patterns were collected from the single acicular phase and binary phase area to confirm the presence of the acicular phase (Figure 3 and Figure 4). Based on the SAED findings, the secondary phase is confirmed as a hexagonal martensite phase α’ with an average diameter of 200 nm.

### 3.2. Mechanical Properties

**Figure 5:**
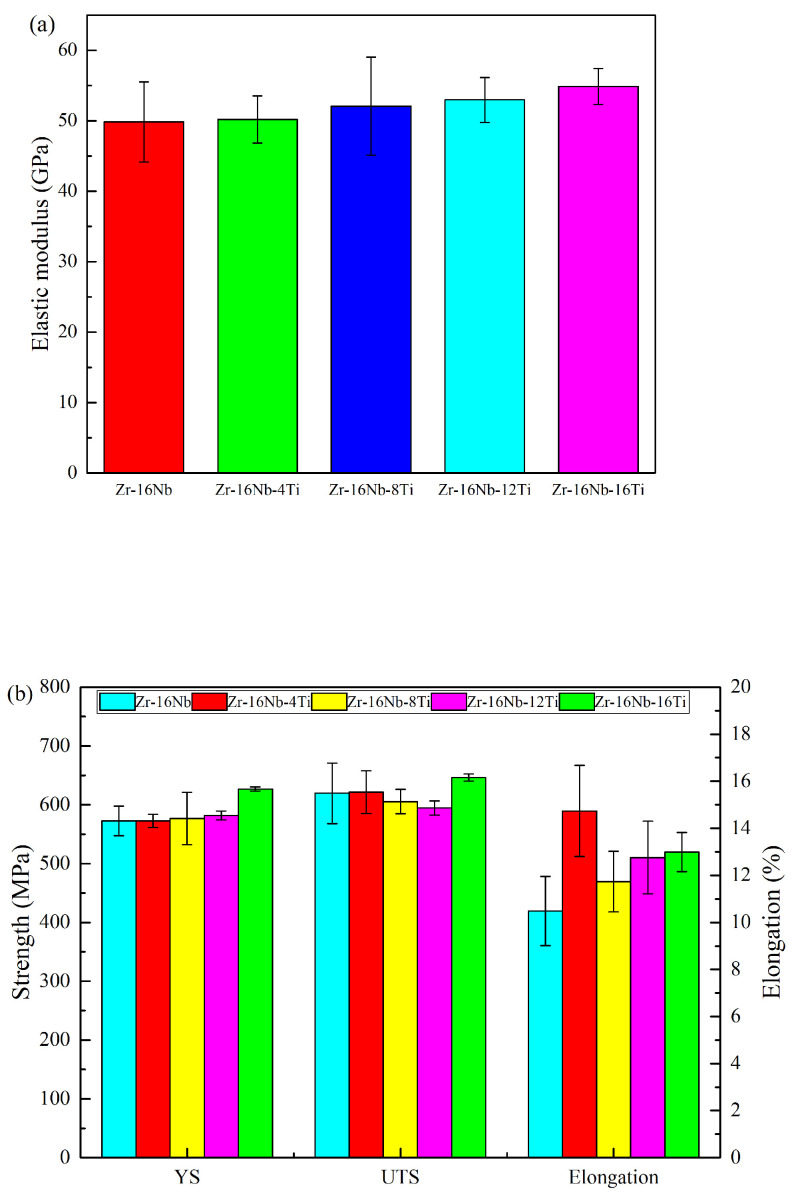
Mechanical properties of the Zr-16Nb-xTi alloys: (**a**) Young’s modulus; (**b**) Strength and Elongation.

The mechanical properties of the Zr-16Nb-xTi alloys are shown in Figure 5. All samples clearly exhibit a Young’s modulus lower than 55 GPa. Zr-16Nb exhibits the lowest modulus of 49.8 GPa, while the value is observed to increase with Ti addition, with Zr-16Nb-16Ti displaying the highest modulus of 54.8 GPa.

Figure 5b illustrates the yield strength (YS), ultimate tensile strength (UTS) and elongation of Zr-16Nb-xTi. YS is noted to gradually increase with Ti addition, with all specimens exhibiting YS values above 572 MPa. Zr-16Nb-16Ti demonstrates the highest YS value of 626.8 MPa. Different characteristics are observed for UTS, which initially increases, followed by a decrease and finally an increase with Ti addition. Zr-16Nb-12Ti exhibits the lowest UTS value of 594.5 MPa, whereas Zr-16Nb-16Ti demonstrates a peak value of 646.2 MPa. With respect to elongation, Zr-16Nb-4Ti displays superior ductility (14.74%), while Zr-16Nb has a minimum value of 0.48%. The other samples display an increase in ductility with Ti addition.

### 3.3. Magnetic Susceptibilities

**Figure 6:**
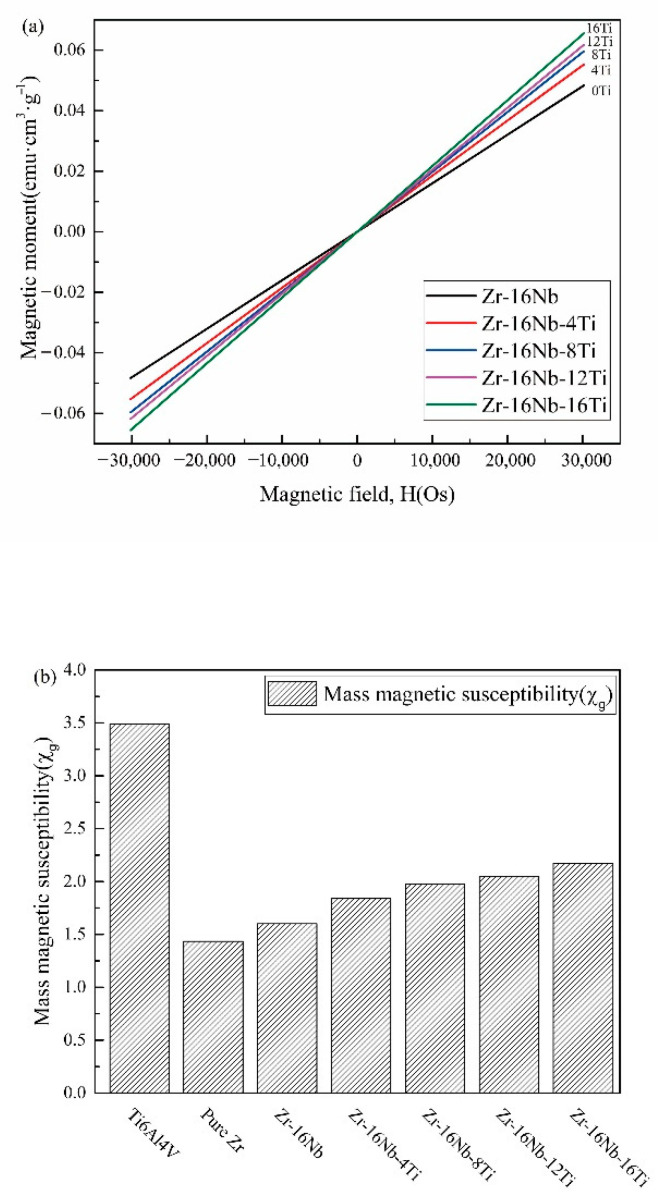
(**a**) Magnetizing curves of Zr-16Nb-xTi and (**b**) magnetic susceptibility of the Zr-16Nb-xTi alloys and contrast samples.

Figure 6 displays the magnetic moment variation of the Zr-16Nb-xTi alloys caused by the magnetic field at room temperature. While changing the magnetic field, the magnetization curve of the Zr-16Nb-xTi alloys is observed to maintain linearity, which indicates that the paramagnetic property of Zr is unchanged when adding alloying elements Nb and Ti. The magnetic susceptibility of the Zr-16Nb-xTi alloys, Ti-6Al-4V and pure Zr, is determined by the slope of the magnetization curve via linear data fitting. As can be seen, the Zr-based alloys exhibit lower magnetic susceptibility than Ti-6Al-4V (3.4874 × 10^−6^ cm^3^/g) and pure Ti (3.2 × 10^−6^ cm^3^/g) [29]. It is worth noting that the susceptibilities of pure Zr (1.4346 × 10^−6^ cm^3^/g) and Zr-16Nb (1.6022 × 10^−6^ cm^3^/g) are less than half of the value of the Ti-6Al-4V alloy. The Zr-16Nb-xTi alloys display enhanced magnetic susceptibility with increasing Ti content, with the highest magnetic susceptibility of 2.17275 × 10^−6^ cm^3^/g observed for the Zr-16Nb-16Ti alloy.

### 3.4. Ion Release

The average ion concentration of Zr, Nb and Ti released from Zr-16Nb-xTi in Hank’s simulated body fluid solution was in the range of 0.001–0.005 mg/L, which was higher than the minimum measurement capability of the equipment (0.001 mg/L). As can be observed from Table 2, adding Nb and Ti to the Zr-based alloys leads to the release of even lower ion concentrations, enough to be detected by the equipment.

### 3.5. Electrochemical Corrosion Properties of the Zr-16Nb-xTi Alloys

**Figure 7:**
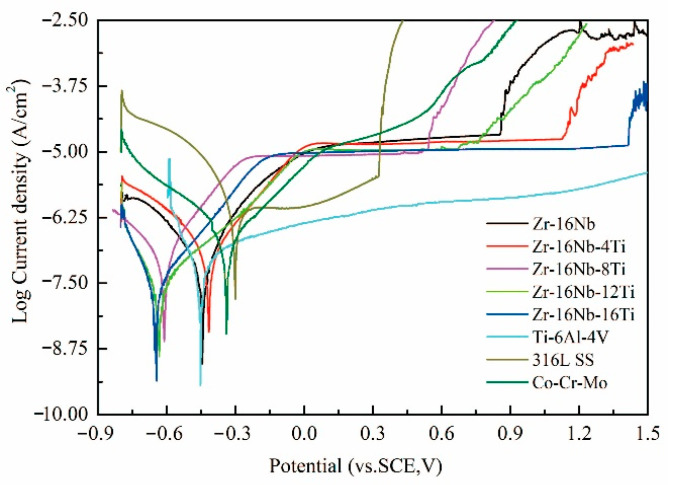
Potentiodynamic polarization curves of the Zr-16Nb-xTi and reference samples.

The potentiodynamic polarization test was employed to analyze the chemical corrosion behavior of the samples. The potentiodynamic polarization curves of the test samples in Hanks’ balanced salt solution (HBSS) at 37 °C are shown in Figure 7. The polarization characteristics leading to passive film formation and growth on the alloy surface are observed to be similar. The potential increases are rapidly followed by transpassivation, which indicates the rapid growth of pitting after the breakdown of the passive film. The average values of the electrochemical parameters of Zr-16Nb-xTi, such as pitting potential (*E*_pit_), corrosion current density (*I*_corr_) and corrosion potential (*E*_corr_), are listed in Table 3, along with those of Ti-6Al-4V, 316L SS and Co-Cr-Mo alloy for comparison.

According to the electrochemical theory, the smaller the self-corrosion current density and the more positive the self-corrosion potential of the alloy, the better its corrosion resistance. The self-corrosion current density plays a key role in reflecting the corrosion resistance of the alloys. As can be observed from Table 3, though Zr-16Nb-12Ti and Zr-16Nb-16Ti have low *E*_corr_ values, their *I*_corr_ values are quite small among the test samples, indicating superior corrosion resistance. 

As can be seen in the passivation region in Figure 7, the primary corrosion mechanism of the alloys is general corrosion owing to the growth of the passive film, with an overall low corrosion rate. The corrosion rate obviously increases with the further enhanced corrosion potential due to the cracking of the passivating film and pitting corrosion. The difference between the pitting and corrosion potentials (Δ*E* = *E*_pit_ − *E*_corr_) and pitting potential are employed to quantitatively analyze the corrosion resistance for a passive film. The pitting potentials for Zr-16Nb-xTi, 316L SS and Co-Cr-Mo alloys are observed to be 0.535–1.367 V, 0.230 V and 0.466 V, respectively, whereas the stable passive states display the potentials of 1.152–1.988 V, 0.536 V and 0.865 V, respectively. Ti-6Al-4V is observed to display no transpassivation until 2 h. It is worth noting that Zr-16Nb-xTi alloys have a larger passive region than the reference alloys, with the widest passive region (Δ*E* = 1.988 V) and highest pitting potentials (*E*_pit_ = 1.367 V) for Zr-16Nb-16Ti suggesting its noble corrosion resistance. 

### 3.6. Assessment of In Vitro Cytocompatibility

**Figure 8:**
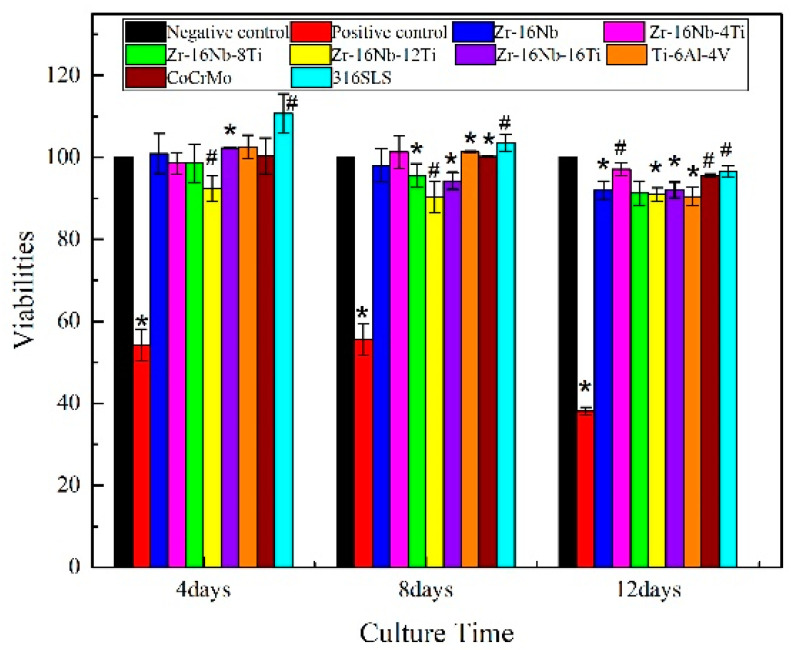
Viabilities of MG63 osteoblast-like cells, which were cultivated in the extracted media of Zr-16Nb-xTi, Ti-6Al-4V, Co-Cr-Mo and 316L SS for 4, 8 and 12 days. #, * indicates *p* < 0.05 and *p* < 0.01, respectively, compared with the negative control.

The relevant viability of the osteoblast-like cells (MG63) cultivated in the extracted media of Zr-16Nb-xTi, Ti-6Al-4V, Co-Cr-Mo and 316L SS for different periods (4, 8 and 12 days) is illustrated in Figure 8. After four days, the cell viability of the alloys is observed to range from 92% (Zr-16Nb-12Ti) to 110% (316L SS), with the cell viabilities of most alloys higher than the negative control group, implying a nontoxic nature. Noticeably, the cell viability of the Zr-16Nb-xTi alloys reduces with increasing Ti; however, the materials still exhibit optimal biocompatibility. After eight days, the cell viability of the alloys is observed to decline, except Zr-16Nb-4Ti, which exhibits a small growth. Overall, the viability values remain in the range of 90%–103%.

**Figure 9:**
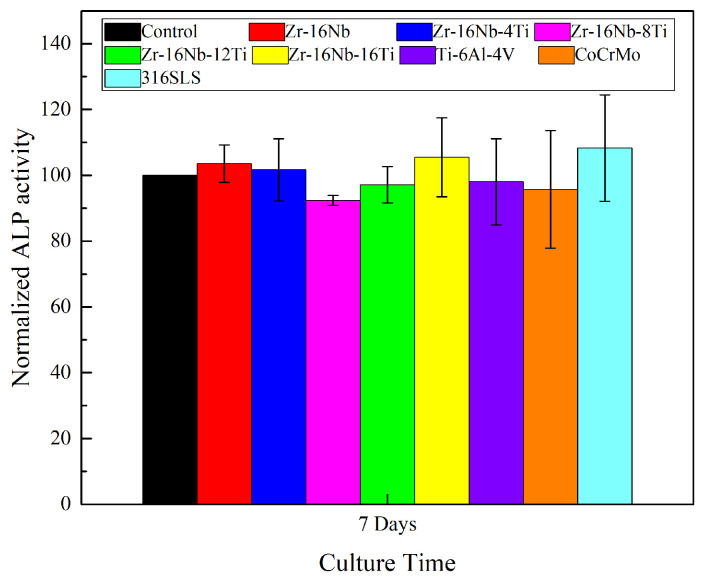
MG 63 cells lysates normalized alkaline phosphatase (ALP) activities after cell cultivating in extracts of Zr-16Nb-xTi, Ti-6Al-4V, Co-Cr-Mo and 316L SS alloys for 7 days.

The normalized ALP activity of the MG63 osteoblast-like cells cultivated in the extraction media for seven days is shown in Figure 9. The ALP activity of the MG63 cells can reflect different osteoblasts functions for the cells in various media. It can be noted from Figure 9 that the ALP activity of most of the alloys lies at the same level as the negative control group, ranging from 92% to 108%. It is worth mentioning that Zr-16Nb, Zr-16Nb-4Ti, Zr-16Nb-16Ti and 316L SS exhibit higher values compared to the control group, indicating excellent osteoblast induction. Despite having the lowest value among the samples, the ALP activity of Zr-16Nb-8Ti is noted to still maintain a high standard (92%).

### 3.7. Blood Compatibility Test

Table 4 illustrates the respective hemolysis rate of the alloys. The rate for the samples is noted to lie at low levels, implying the negligible effect of the samples on the red blood cells. It is also observed that the rate of pure Ti is higher compared to the other alloys and is nearly twice that of pure Zr. In addition, the rate of Zr-16Nb-xTi is noted to increase until 8%Ti is added, followed by a decrease. In general, the Zr-based alloys have a lower rate than Ti and represent superior hemocompatibility.

According to ASTM F756-00, the hemolysis ratio among 0–2% is nonhemolytic, while 2–5% indicates mild hemolysis. It is hemolysis when the ratio is beyond 5% [30].

**Figure 10:**
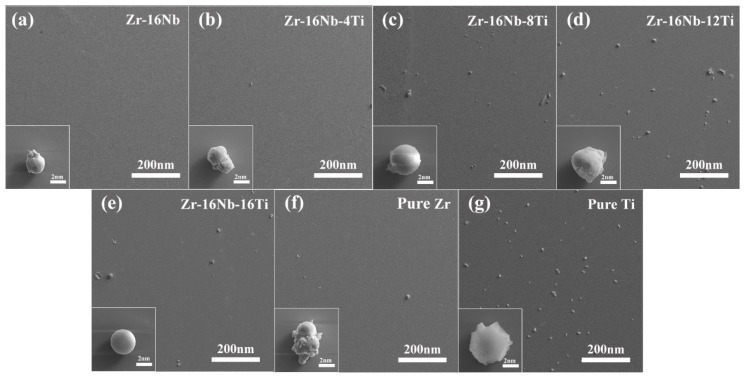
Platelet adhesion situation on the Zr-16Nb-xTi and reference alloys under the magnification times of 60× (inset is the morphology of platelets under 2000× times magnification): (**a**) Zr-16Nb; (**b**) Zr-16Nb-4Ti; (**c**) Zr-16Nb-8Ti; (**d**) Zr-16Nb-12Ti; (**e**) Zr-16Nb-16Ti; (**f**) Pure Zr; (**g**) Pure Ti.

As shown in Figure 10, the platelet adhesion test reveals significant differences in the amounts of platelets adhered to different alloys. Significant platelet adhesion is observed on the surface of pure Ti, whereas only a few platelets adhere to pure Zr. The number of adhered platelets increases with the Ti content in the Zr–16Nb–xTi alloys. Thus, compared with Zr, Ti is observed to attract significant platelet adhesion, which may lead to the clotting phenomenon.

Figure 10 also demonstrates the respective adhesive behavior of the different alloys. The platelets are observed to assume a complete spherical state on the surface of Zr–16Nb, Zr–16Nb–8Ti and Zr–16Nb–16Ti, with no pseudopod seen on the surface, implying difficult platelet aggregation as well as clotting phenomenon. Notably, slight platelet aggregation is observed on the surface of pure Zr, whereas the platelets on the pure Ti surface appear to have lysed, appearing to be flat. Zr–16Nb–4Ti and Zr–16Nb–12Ti also roughly indicate the spherical state of the platelets.

## 4. Discussion

### 4.1. Microstructure and Mechanical Properties of the Zr-16Nb-xTi Alloys

The Young’s modulus is related to the phase constitution, following the order *E*_ω_ > *E*_α’_ > *E*_β_. At the same time, the alloying elements also affect the Young’s modulus, attributed to their inherent mechanical properties, especially evident for the alloys with the same phase constitution. 

In this study, the Zr-16Nb-xTi alloys have the same phase constitution of the β + α’ phase. The only exception is the lower content of the α’ phase in Zr-16Nb compared with other alloys. The martensite α’ phase formed during quenching and the differences in behavior can be attributed to the Ti content. Based on the XRD and TEM results, no ω phase was observed in the Zr-16Nb-xTi alloys developed in this study. This result aligns well with what we stated in the Introduction section. The phase constitution of the Zr-Nb alloy was affected by Ti addition. Studies have reported that Zr prevents martensite formation in Ti-Nb alloys (by reducing the Ms temperature) [22,23] and can be visualized as a β stabilizing element in Ti-Nb systems. Thus, as Ti is added to Zr-Nb systems, it may act as an α stabilizing element, leading to the detection of no ω phase.

As the Zr-16Nb-xTi alloys have the same phase constitution, Ti takes the place of Zr as it has a higher Young’s modulus. The strength of Zr-16Nb-xTi increases with the addition of Ti because of the solution strengthening effect.

### 4.2. Magnetic Susceptibilities of the Zr-16Nb-xTi Alloys

The alloy magnetic susceptibility relates to its alloying elements and phase constitution [31]. Zr has much lower magnetic susceptibility (1.4346 × 10^−6^ cm^3^/g) than Ti (3.2 × 10^−6^ cm^3^/g) [29]. Among various β stabilized elements, Nb possesses low magnetic susceptibility (2.2 × 10^−6^ cm^3^/g) [29] and excellent biocompatibility. As can be seen in Figure 6, the magnetic susceptibility of the Zr-16Nb-xTi alloys changed with Ti addition. As compared with pure Zr, Zr-16Nb displays a small increase in magnetic susceptibility; however, the increase is more obvious with Ti addition. Based on Collings et al.’s [31] research, the constituent phases and their volume fraction had an effect on the magnetic susceptibility of materials. The magnetic susceptibility of the Zr-16Nb-xTi alloys can be expressed as below:

(1) $$\chi_{Zr-16Nb-xTi}=\chi_{\beta }\cdot V_{\beta }+\chi_{\alpha^{\prime }}\cdot V_{\alpha^{\prime }}+\chi_{\omega }\cdot V_{\omega }$$

where $\chi_{\beta }$, $\chi_{\alpha }$ and $\chi_{\omega }$ represent the magnetic susceptibility of the β, α′ and ω phases, respectively, while $V_{\beta }$, $V_{\alpha }$ and $V_{\omega }$ indicate the volume fraction of the β, α′ and ω phases. 

Based on the findings from XRD and TEM, the Zr-16Nb-xTi alloys dominantly contained β and α′ phases. Furthermore, a small extent of the ω phase was present in Zr-16Nb. Thus, the magnetic susceptibility changes could be attributed to the composition of the alloying elements. On account of the higher magnetic susceptibility that Ti possessed, the magnetic susceptibility of Zr-16Nb-xTi increased gradually. The entire magnetic susceptibilities of the alloys were not simply determined by the addition of the respective values of the alloying elements (such as Ti, Nb, etc.). Crystal structure defects (such as vacancies, dislocations and internal stresses) might have an effect on the magnetic susceptibility of alloys [32].

The magnetic susceptibility values of the Zr-Nb alloys subjected to different heat treatments or hot working processes in comparison with other typical implant biomaterials are presented in Table 5. The microstructure and texture can impact the magnetic susceptibility as well as phase constitution or composition. Though Zr-16Nb has the same composition, it displays different magnetic susceptibilities under various conditions, and as $\chi_{\beta }>\chi_{\alpha^{\prime }}>\chi_{\omega }$, we can adjust the magnetic susceptibility via heat treatment or hot working processes to obtain the required phase and texture. Compared with Ti-6Al-4V and Co-Cr-Mo, Zr-Nb and Zr-16Nb-xTi alloys possess much lower magnetic susceptibility, indicating the impairing of the artifact effect during MRI testing.

### 4.3. In Vitro Cytocompatibility Assessment of the Zr-16Nb-xTi Alloys

As shown in Figure 8 and Figure 9, the Zr-16Nb-xTi alloys exhibit excellent cytocompatibility compared with the typical traditional biomedical alloys (Ti-6Al-4V, Co-Cr-Mo and 316L SS), which can be attributed to the outstanding biocompatibility of Zr, Nb and Ti. Eisenbarth et al. [12] mentioned the biocompatibility of the main alloying element in Ti alloys, such as Nb, Mo, Ta, Zr and Al. It was observed based on a series of in vivo and in vitro experiments that Nb exhibited the highest biocompatibility among these elements. Khan et al. [33] and Semlitsch et al. [34] also reported that Nb had outstanding short-term and long-term biocompatibility. Eisenbarth et al. [12] reported that the biocompatibility of alloying elements can be classified as follows: Nb > Ta > Ti/Zr > Mo. In the current study, the cell experiments confirmed the superior cytocompatibility of Zr-16Nb-4Ti, though it was slightly lower than 316L SS with respect to cell viability and ALP activity. Kumar et al. [35] reported the density of cells on the coated TNZ specimen was superior to the uncoated one. It was noted that the cells cultured on the PEDOT/FHA specimens had a polygonal shape and were consistently spread across the coating on the substrate, indicating that the surface modification can improve the bioactivity of alloys. Thus, the samples developed in the current study can be further studied in areas such as surface modification and in vivo implant experiments for comprehensive biocompatibility assessment.

### 4.4. Corrosion Properties of the Zr-16Nb-xTi Alloys

It can be seen in Figure 7 that the obvious characteristics of the anodic polarization curves of the Zr-16Ni-xTi alloys were the stable passive region and spontaneous passivation behavior. In the stable passive region, the current changes slightly with the increase of voltage due to the protective action of the passive film. However, when the passive film was broken, the pits on the film were easier to nucleate and grow. Based on the electrochemical theory, in this case, the corrosion resistance of the samples depends largely on the stability and resistance of the passive film. In addition, the difference between the corrosion potentials and pitting (Δ*E* = *E*_pit_ − *E*_corr_) is usually used to analyze the stability and passive film resistance [36].

Therefore, it could be inferred that the variation in the corrosion resistance of the Zr-16Ni-xTi alloys was attributable to the changes in the composition and structure of the passive film. Based on the literature studies, Zr, Nb and Ti metals are observed to spontaneously oxidize in the air or aqueous solutions to form ZrO_2_, Nb_2_O_5_ and TiO_2_ (passive film) on the alloy surfaces, respectively [21]. Thus, the mixed oxides of ZrO_2_-Nb_2_O_5_-TiO_2_ might form on the alloy surfaces after Ti doping instead of ZrO_2_-Nb_2_O_5_. The difference in the Ti content changed the composition of the passivation film on the Zr-16Ni-xTi alloys, affecting its stability, which changed the corrosion resistance of the samples. The widest passive region (Δ*E* = 1.988V) and highest pitting (*E*_pit_ = 1.367 V) potentials of Zr-16Nb-16Ti noted from Table 3 suggested the noble corrosion resistance. It can be postulated for the Nb content reaching 16 (wt.%) that the passivation film on the alloy surface exhibits optimal stability and resistance. In addition, the broken potential (*E*_pit_) of the Zr-16Ni-xTi alloys was, on average, 0.535–1.367 V, which was higher than the body potential corresponding to the oxidation-reduction potential of the body fluid (0.4–0.5 V) [12]. The excellent corrosion resistance was aligned well with the ion release experiment result. Zr ion concentration, released from pure Zr and Zr-16Nb-xTi alloys, were extremely low, which can reach the minimum detection limit of the ICP equipment, in comparison with pure Ti.

Therefore, the Zr-16Ni-xTi alloys can remain in the passive state after being implanted in the body, which reduces the corrosion rate, exhibiting a strong prospect for the development of implants.

### 4.5. Blood Compatibility Test

Blood compatibility is an important indicator of implant biomaterials. Immune responses as well as chemical, physical and other factors can cause the hemolytic reaction of red blood cells. 

In hemolysis, as the biological material comes in contact with the blood, the red blood cells are damaged to varying degrees, releasing hemoglobin. The degree of damage to the red blood cells reflects the hemolytic rate. Based on the hemolysis rate test, the values of the Zr-16Nb-xTi samples are far smaller than 5% and even less than 0.3% in some cases. Noticeably, the rate of pure Zr is less than half of pure Ti, which represents the main reason for the optimal hemocompatibility of Zr-16Nb-xTi. 

Goodman et al. [37] reported that the platelet morphology could be divided into five categories: (a) discoid or round, (b) dendritic, (c) early pseudopodial, spread dendritic, (d) intermediate pseudopodial, spreading and (e) fully spread. As shown in Figure 10, the platelets are dispersed with no obvious sign of accumulation on the test materials. In addition, it can be observed that the platelets maintain a spherical shape on the surfaces of the Zr-16Nb-xTi alloys. On the other hand, the platelets assume an incomplete spherical morphology on pure Ti, and the platelets are noted to be activated with a slight pseudopodia extension on the surface of pure Zr. Wang et al. [38] reported that the degree of roughness has a remarkable effect on platelet activation. In this research, noticeably severe platelet activation was detected with clear pseudopodia extension and aggregation on the rough surface, indicating the high possibility of a hemolytic phenomenon. For implant biomaterials, a decrease in the surface roughness should be achieved to avoid the hemolytic phenomenon. In the current study, the samples were polished before the hemolysis test to maintain a smooth state. The platelets maintained the spherical shape and did not undergo aggregation or activation, exhibiting superior hemocompatibility.

## 5. Conclusions

Zr-16Nb-xTi alloys were prepared in this study along with the characterization of their microstructure, mechanical properties, magnetic susceptibility and in vitro biocompatibility to evaluate the potential of use as biomedical implant materials.

1. The martensite α’ phase was formed in the Zr-16Nb-xTi alloys during the quenching process. The content of the α’ phase in Zr-16Nb was observed to be less than the other samples via TEM; however, the determination of their accurate constitution needs further research. The average Young’s modulus of Zr-16Nb-xTi (52 GPa) was lower than most of the Ti-based alloys. The Zr-16Nb-xTi alloys exhibited much lower magnetic susceptibility as compared to Ti-6Al-4V because of the presence of the martensite α’ phase caused by Ti addition and the lower magnetic susceptibility of Zr. It is suggested that the α’ + β phase is suitable for achieving a low modulus and magnetic susceptibility.
2. Immersion in Hank’s simulated body fluid for 30 days exhibited a very low level of the ion release from Zr-16Nb-xTi. The observed results aligned well with the findings from the electrochemical corrosion behavior test. The addition of Ti enhanced the corrosion resistance of Zr-16Nb-xTi, with Zr-16Nb-16Ti exhibiting a small *I*_corr_ value and the widest passivation region, demonstrating the best corrosion resistance among the test samples.
3. The MG63 cells retained high viability (91% to 97%) after cultivation in the extraction media of Zr-16Nb-xTi for 12 days. The hemolysis rate for the Zr-16Nb-xTi value was observed to be less than 0.3%. The SEM analysis revealed that the platelets maintained spherical shape and did not undergo aggregation or activation, displaying superior hemocompatibility.

## Figures and Tables

**Table 1:** Chemical compositions of the Zr-16Nb-xTi alloys determined by EDS (wt.%).

Element	Zr-16Nb	Zr-16Nb-4Ti	Zr-16Nb-8Ti	Zr-16Nb-12Ti	Zr-16Nb-16Ti
Zr	83.453	79.587	75.770	71.444	67.012
Nb	16.547	16.370	16.323	16.398	16.563
Ti	-	4.043	7.907	12.158	16.425

**Table 2:** Zr, Nb, Ti ion concentration release from pure Zr, pure Ti and the Zr-16Nb-xTi alloys.

Samples	Nb (mg/L)	Ti (mg/L)	Zr (mg/L)
Zr-16Nb	0.001	-	0.002
Zr-16Nb-4Ti	0.005	0.001	0.002
Zr-16Nb-8Ti	-	-	0.002
Zr-16Nb-12Ti	0.002	-	-
Zr-16Nb-16Ti	-	0.002	-
Pure Zr	-	-	-
Pure Ti	-	0.010	-

The “-” in the table indicates that it was not detected.

**Table 3:** Corrosion parameters of the Zr-16Nb-xTi and reference alloys.

Samples	OCP (V)	*E*_corr_ (V)	*I*_corr_ (10^−8^/cm^2^)	*E*_pit_ (V)	Δ*E*
Zr-16Nb	−0.417 (0.045)	−0.414 (0.045)	14.76 (3.059)	0.88 (0.028)	1.294
Zr-16Nb-4Ti	−0.492 (0.121)	−0.409 (0.084)	5.075 (2.434)	1.15 (0.0707)	1.559
Zr-16Nb-8Ti	−0.591 (0.087)	−0.617 (0.062)	6.461 (3.068)	0.535 (0.021)	1.152
Zr-16Nb-12Ti	−0.564 (0.028)	−0.620 (0.017)	2.070 (0.220)	0.722 (0.092)	1.342
Zr-16Nb-16Ti	−0.610 (0.032)	−0.621 (0.035)	2.373 (1.426)	1.367 (0.153)	1.988
Ti-6Al-4V	−0.247 (0.014)	−0.388 (0.093)	4.501 (1.650)	-	-
316L SS	−0.284 (0.024)	−0.306 (0.144)	68.729 (11.634)	0.230 (0.134)	0.536
Co-Cr-Mo	−0.257 (0.017)	−0.399 (0.087)	16.610 (2.927)	0.466	0.865

**Table 4:** Hemolysis rate of testing alloys.

Samples	Hemolysis Ratio	Hemolytic Standard
Zr-16Nb	0.156%	Nonhemolytic
Zr-16Nb-4Ti	0.207%	Nonhemolytic
Zr-16Nb-8Ti	0.255%	Nonhemolytic
Zr-16Nb-12Ti	0.219%	Nonhemolytic
Zr-16Nb-16Ti	0.170%	Nonhemolytic
Pure Zr	0.161%	Nonhemolytic
Pure Ti	0.326%	Nonhemolytic

**Table 5:** Magnetic susceptibility of the Zr-Nb alloys and typical implant biomaterials.

Samples	Phase Constitution	$\mathrm{Magnetic}\;\mathrm{Susceptibility},\;\chi_{g}\;(\times 10^{-6}\;{\mathrm{cm}}^{3}/g)$
Zr-3Nb (as-cast) [6]	α′	~1.13
Zr-6Nb (as-cast) [6]	β, α′, ω	~1.15
Zr-9Nb (as-cast) [6]	β, ω	~1.17
Zr-12Nb(as-cast) [6]	β, ω	~1.28
Zr-14Nb (as-cast) [6]	β, ω	~1.45
Zr-14Nb (90% cold-rolled) [7]	β, ω	~1.49
Zr-16Nb (as-cast) [6]	β, ω	~1.50
Zr-16Nb (solution and quenching) [7]	β, ω	~1.38
Zr-16Nb (573 K aging) [7]	β, ω, α	~1.19
Zr-22Nb (as-cast) [6]	β	~1.45
Zr-1Nb (annealed) [8]	α	1.28
Zr-16Nb (in this work)	β, ω	1.60
Zr-16Nb-4Ti (in this work)	β, α′	1.83
Zr-16Nb-8Ti (in this work)	β, α′	1.97
Zr-16Nb-12Ti (in this work)	β, α′	2.04
Zr-16Nb-16Ti (in this work)	β, α′	2.17
Ti-6Al-4V (in this work)	-	3.47
Co-Cr-Mo [7]	-	~7.5
